# Sexual Dysfunction among Female Patients of Reproductive Age in a Hospital Setting in Nigeria

**Published:** 2007-03

**Authors:** Benjamin A. Fajewonyomi, Ernest O. Orji, Adenike O. Adeyemo

**Affiliations:** ^1^ Department of Community Health; ^2^ Department of Obstetrics and Gynaecology; ^3^ Department of Medicine, Faculty of Clinical Sciences, Obafemi Awolowo University, Ile-Ife, Nigeria

**Keywords:** Sexual dysfunction, Sexuality, Cross-sectional studies, Nigeria

## Abstract

Although sexual dysfunction is an important public-health problem in Nigeria, little research has been conducted on this topic in Nigeria. This cross-sectional study was conducted to determine the prevalence of sexual dysfunction and their correlates among female patients of reproductive age using a questionnaire. Respondents were recruited from the out-patients clinics of a teaching hospital setting in Ile-Ife/Ijesa administrative health zone, Osun State, Nigeria. Of 384 female patients interviewed, 242 (63%) were sexually dysfunctional. Types of sexual dysfunction included disorder of desire (n=20; 8.3%), disorder of arousal (n=13; 5.4%), disorder of orgasm (n=154; 63.6%), and painful coitus (dyspareunia) (n=55; 22.7%). The peak age of sexual dysfunction was observed among the age-group of 26–30 years. Women with higher educational status were mostly affected. The reasons for unsatisfactory sexual life mainly included psychosexual factors and medical illnesses, among which included uncaring partners, present illness, excessive domestic duties, lack of adequate foreplay, present medication, competition among wives in a polygamous family setting, previous sexual abuse, and guilt-feeling of previous pregnancy termination among infertile women. The culture of male dominance in the local environment which makes women afraid of rejection and threats of divorce if they ever complain about sexually-related matters might perpetrate sexual dysfunction among the affected individuals. Sexual dysfunction is a real social and psychological problem in the local environment demanding urgent attention. It is imperative to carry out further research in society at large so that the health and lifestyles of affected women and their partners could be improved.

## INTRODUCTION

Sexual dysfunction is a group of disorders associated with desire, arousal, orgasm, and painful sex (dyspareunia and vaginismus) (1). Sexual intercourse is as old as humanity itself and is necessary for the propagation of the species. Sexual intercourse is not only influenced by the integrity of the genital tract but also by the limbic system and spinal arousal centres (2–3). A large component of sexual desire in women is responsive rather than spontaneous. Therefore, motivation and ability of women to find and respond to sexual arousal and subsequent sexual desire is crucial, but complex. In ongoing relationships, motivation of a woman appears to be largely influenced by her intimacy with her partner and her wish to enhance it. It correlates well with how mentally-exciting she finds the sexual stimulus and its context and poorly with objective genital blood flow changes (4–7).

Epidemiological investigations of women with sexual dysfunction from well-designed randomly-sampled community-based population are limited. Available information shows that female sexual dysfunction is common and occurs in 22–43% of women (4) and 30–50% of American women (5–6). The prevalence rates in Africa, especially in Nigeria, are either non-existent or scarce. The aetiology of sexual dysfunction is varied and results from a complex interaction of biological, psychological and social factors (7). Psychological causes may include anger, depression, anxiety, ignorance, or deeper psychological conflicts. Interpersonal factors involve conflicts with the partner or an inability to establish interpersonal relationship or divorce. Physical causes include illness (for instance breast cancers, infertility), injury, or drugs (for instance sedative drugs). Sexual function can also be strongly influenced by one's own sense of self and social competence, level of education, vaginal atrophy associated with declining oestrogen levels at menopause, or relative vaginal dryness in early postpartum period (8–17).

Sexual dysfunction severely affects the quality of life of patients, but studies in Nigeria are scarce. This study was, therefore, conducted to determine the prevalence of sexual dysfunction and their correlates among female patients of reproductive age at Obafemi Awolowo University Teaching Hospital Complex, Ile-Ife, Nigeria.

## MATERIALS AND METHODS

This is a cross-sectional survey of female patients of reproductive age attending the gynaecological, medical, surgical, psychiatric and general out-patient clinics at Obafemi Awolowo University Teaching Hospitals Complex, Ile-Ife. Patients were all educated on the sensitive nature of the study, and only consenting women were recruited into the study.

The sample size for the study was calculated using the formula below according to Araoye (18), assuming the prevalence rate of 50% for sexual dysfunction in this environment:

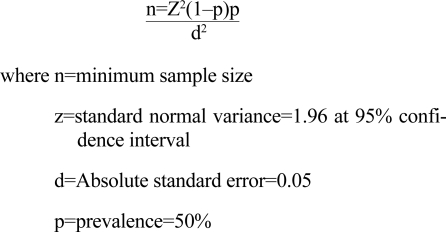


Using the above formula, the calculated sample size was approximately 384. Three hundred and eighty four clients were, therefore, recruited.

A set of pretested structured questionnaire, designed by the authors, was used for collecting information. The questionnaire contained both open- and close-ended questions. The interviewers were final-year female students who had been properly trained on the sensitive nature of the study. There was no inducement to patients to participate in the study, and there was no denial or punishment for refusal to participate. Only those who gave consent after detailed counselling were recruited. These patients were also told that the investigation is entirely for research purposes. The name, hospital number, and addresses of the patient were not recorded to ensure confidentiality of information given. A female student privately interviewed each respondent. The set of questionnaire was translated into the local dialect for non-literate patients.

Female sexual dysfunction in this study was defined as women of reproductive age, who experienced any of the disorders associated with desire, arousal, orgasm, and painful sex (dyspareunia and vaginismus) in the three months preceding the study. A pilot study was done in a separate hospital setting, and relevant modifications were made in the questionnaire before commencement of the study.

Section I of the questionnaire elicited information on the sociodemographic background of the respondents. Section 1I investigated their sexual history with respect to sexual desire, arousal, orgasm, sexual pain disorders, emotional state, and the primary problems which brought them to hospital. Information was collated and fed into PC. Data were analyzed using the SPSS softwere (version 11). Description tables and inferential chi-square tests were used where relevant with statistical significance set at p<0.05. The study was conducted during April-June 2002.

The protocol for this study was approved by the Ethical Board of the Obafemi Awolowo University Teaching Hospital Complex, Ile-Ife, Nigeria. The study participants also gave informed consent after the details of the study were explained to each of them and voluntary participation solicited.

## RESULTS

Three hundred and eighty-four female patients of reproductive age were interviewed. Table 1 shows that 63 (16.4%) female patients were in the age-group of 21–25 years, 123 (32.1%) in the age-group of 26–30 years, 86 (22.4%) in the age-group of 31–35 years, 57 (14.8%) in the age-group of 36–40 years, and 55 (14.3%) in the age-group of 41–45 years. Most (83.6%) of them were married, 39 (10.2%) were single, 15 (3.9%) were separated, and nine (2.3%) were divorced. The majority (60.7%) had monogamous family, while 111 (28.9%) were polygamous and 40 (10.4%) were co-habiting. Yoruba (n=326; 84.9%) was the predominant tribe, followed by Igbo (n=10; 2.6%), and Hausa (n=6; 1.6%), and 42 (10.9%) belonged to other ethnic groups.

**Table 1.** Selected sociodemographic and biological variables

Variable	No.	Percentage
Age-group (years)		
21–25	63	16.4
26–30	123	32.1
31–35	86	22.4
36–40	57	14.8
41–45	55	14.3
Level of education		
None	28	7.3
Primary	54	14.1
Secondary	135	35.2
Tertiary	167	43.3
Marital status		
Married	321	83.6
Single	39	10.2
Separated	15	3.9
Divorced	9	2.3
Family setting		
Monogamous	233	60.7
Polygamous	111	28.9
Co-habiting	40	10.4
Ethnicity		
Yoruba	326	84.9
Ibo	10	2.6
Hausa	6	1.6
Others	42	10.9
Sexually dysfunctional		
Yes	242	63.2
No	142	36.7
Types of sexual dysfunction (n=242)		
Disorder of desire	20	8.3
Disorder of arousal	13	5.4
Disorder of orgasm	154	63.6
Dyspareunia (painful coitus)	55	22.7
Reasons for sexual dysfunction (n=242)*		
Uncaring partner	197	81.4
Inadequate foreplay	80	33.1
Competition among wives in a polygamous family	80	33.1
Present illness	45	18.5
Guilt feeling from previous pregnancy terminations	45	18.5
Lack of interest	43	17.8
Present medication	22	9.1
Excessive homework	15	6.2
Dyspareunia	15	6.2

*Most patients gave multiple reasons

One hundred and sixty seven (43.4%) of the 384 female patients had tertiary level of education, 135 (35.2%) had secondary education, 54 (14.1%) had primary education, and 28 (7.3%) had no formal education. Although 242 (63%) women had sexual dysfunction, the majority (37%) were not sexually dysfunctional. Types of sexual dysfunction included disorder of desire (n=20; 8.3%), disorder of arousal (n=13; 5.4%), disorder of orgasm (n=154; 63.6%), and painful coitus (dyspareunia) (n=55; 22.7%). The reasons for sexual dysfunction among those involved included uncaring partner (n=197; 81.4%), inadequate foreplay (n=80; 33.1%), present illness (n=45; 18.5%), followed by lack of interest (n=43; 17.8%), present medication (n=22; 9.1%), excessive homework (n=15; 6.2%), dyspareunia (n=15; 6.2%), competition among wives in a polygamous family setting (n=15; 6.2%), previous sexual abuse (n=80; 33.1%), and guilt-feeling of previous pregnancy termination among infertile women (n=45; 18.5%). Some patients gave multiple reasons.

Table 2 shows the relationship between sexual dysfunction and selected variables. Various illnesses, such as medical, surgical, psychiatric, and gynaecological problems, were significantly associated with sexual dysfunction (p<0.001). Sixty (75%) of the 80 respondents who volunteered to disclose history of sexual abuse were sexually dysfunctional (p<0.001). The polygamous family type was likely to be sexually dysfunctional (p=0.02). Emotionally-unstable women were highly associated with sexual dysfunction (p< 0.001).

**Table 2.** Relationship between sexual dysfunction and selected variables

Variable	Sexually dysfunctional	Not sexually dysfunctional	p value
Various illnesses		20	0.001
Medical illness	63	24	significant
Surgical illness	41	22	
Psychiatric illness	60	26	
Gynaecologic illness	78	26	
Sexual abuse			
Sexually abused	60	20	<0.001
Not sexually abused	82	122	significant
Type of family			
Monogamous	148	121	0.02
Polygamous	68	38	significant
About to marry	26	12	
Emotional state			
Emotionally stable	45	92	<0.001
Emotionally unstable	197	45	significant
Irritable	100	20	
Depression	70	15	
Family quarrel	27	10	

## DISCUSSION

The findings of the study suggest that female sexual dysfunction is a significant problem that affects a substantial number of women in this environment. The prevalence of sexual dysfunction among women of reproductive age in this study was 68.3%, which is higher than various figures of 22–43% (4) and 30–50% (5) reported in different American populations. The higher prevalence in this study is surprising considering the fact that these patients did not primarily present with sexually-related problems. The obvious reasons for this high prevalence are not known but various reasons may be adduced. First, in Nigeria, many obstacles prevent women from expressing their views about sex and sexual matters. The culture of male dominance often makes them afraid of rejection and threats of divorce if they ever complain about sex-related matters; hence, many women suffer in silence. Second, even when men and women discuss sexual issues, it is often not on equal terms (19–21). A third reason may relate to virtual absence of sexual disorders clinics in our environment. As such, women with such complaints have no avenues to channel these, and they rather prefer remaining in silence, more so in cultures which view sexual matters as sacred (20).

Different reproductive age-groups were affected; however, the peak age of sexual dysfunction in this study was among women aged 26–30 years. This differs from the observation from other reports that female sexual dysfunction increases with increasing age (3, 4, 6, 8). This may be due to the population studied which was predominantly female patients of reproductive age. The increase in sexual dysfunction with increasing age had been attributed to the changes in hormonal status during menopause (9). This is due to the decline in circulatory oestrogen level which leads to varying degrees of vaginal atrophy. It has also been noted that age-related physiological changes do not render a meaningful sexual relationship impossible or even necessarily difficult. The extent to which aging affects sexual function depends largely on psychological, pharmacological, and illness-related factors (10, 11).

In general, female populations with higher educational levels had been associated with less sexual dysfunction (6, 12). In this hospital-based study, sexual dysfunction was highest among women with tertiary education (40.1%) and lowest among women with no education (8%). This may be explained by the fact that, in our environment, it is the highly-educated women who used hospital services more than the uneducated. For instance, in this study, 43.4% of the women had tertiary education compared to 7.3% with no formal education. The higher prevalence of sexual dysfunction among the highly-educated women may also result from the fact that they are bolder to discuss sexual matters openly. In short, it has been documented that the higher the level of female education the higher the probability that they can discuss reproductive health issues with men (19, 20).

In this study, the reasons given by 68.3% of the respondents for unsatisfactory sexual life mainly included uncaring partner (81.8%), followed by inadequate foreplay, present illness, lack of interest, present medication, excessive home work, and dyspareunia. Uncaring partners cited as a major reason demands attention because a large component of sexual desire of women is responsive rather than spontaneous. Motivation of woman appears to be largely influenced by her emotional intimacy with her husband (6, 13, 14). Unfortunately, the patriarchal system of living in Nigeria which encourages male dominance in reproductive matters, including when to have sexual intercourse or not with their wives, does not recognize the emotional intimacy of sexual response in women. Evidence showed that women had little control over their sexual life (19–22). More than eight in every ten women claimed not to have any control at all (21). In Nigeria, the situation of women is worsened by the fact that some traditional norms tend to sanction behaviour of men and make women more sexually submissive and less assertive. These include the stigma of divorce, the culture of total submission to husband who is the head of the family, etc. (22). The implication is that our women are subjected to sex at any time without restriction. It is, therefore, not surprising that inadequate foreplay is another major reason given for sexual dysfunction. The fears of social consequences (being beaten, divorced/abandoned, neglected, etc.) tend to take priority over the fears of the health consequences of such ill-timed sexual acts (19, 22).

Present illness (12, 15), previous history of sexual abuse (16), living in polygamous family type, and emotionally-unstable individuals are significantly at increased risk of sexual dysfunction. The emotional and psychological trauma of previous sexual abuse appears to play a role among in sexual dysfunction among some survivors of sexual abuse (16). Guilt-feelings connected with previous abortion seem to torture infertile women and also various pressures from in-laws and relatives (17). Drugs that have central stimulating action have depressant effect on libido or arousal and, therefore, cause sexual dysfunction (12, 15).

The findings of this study are a pointer to the need for establishment of sexuality disorder clinics in our environment. This should be a multi-disciplinary clinic, incorporating sex therapist, psychologist, gynaecologist, urologist, psychiatrist, social workers, and other trained nursing personnel. A detailed multi-disciplinary history and physical examinations are necessary in all cases to identify the specific disorder present in each individual. This will help individualize the therapy. Drugs, including androgen replacement therapy, aimed at increasing spontaneous sexual desire of women or their arousability, may have a role if other psychological factors affecting arousability are addressed in tandem (12). Low-dose oestrogen therapy may benefit some menopausal women.

In conclusion, observations from this study indicate that sexual dysfunction may be an important public-health problem in Nigeria which has not been investigated. The underlying gender inequality (23) in Nigeria may be one significant reason while those affected remain silent. It is recommended that sexual dysfunction should be seen as an important health problem, and a broader-based study at the community level needs to be carried out to further elucidate the cause, effect, and magnitude of these problems among couples in the African setting. It is also necessary to establish sexual disorders clinics in our environment so that affected individuals will benefit from current treatment options. A multi-disciplinary team approach is required for optimal management.
